# Motor and cognitive growth following a Football Training Program

**DOI:** 10.3389/fpsyg.2015.01627

**Published:** 2015-10-27

**Authors:** Marianna Alesi, Antonino Bianco, Johnny Padulo, Giorgio Luppina, Marco Petrucci, Antonio Paoli, Antonio Palma, Annamaria Pepi

**Affiliations:** ^1^Department of Psychology and Educational Science, University of PalermoPalermo, Italy; ^2^eCampus UniversityNovedrate, Italy; ^3^Department of Biomedical Sciences, University of PaduaPadua, Italy

**Keywords:** children, Football Exercise Program, motor skills, visual selective attention, visual discrimination

## Abstract

Motor and cognitive growth in children may be influenced by football practice. Therefore the aim of this study was to assess whether a Football Training Program taken over 6 months would improve motor and cognitive performances in children. Motor skills concerned coordinative skills, running, and explosive legs strength. Cognitive abilities involved visual discrimination times and visual selective attention times. Forty-six children with chronological age of ∼9.10 years, were divided into two groups: Group 1 (*n* = 24) attended a Football Exercise Program and Group 2 (*n* = 22) was composed of sedentary children. Their abilities were measured by a battery of tests including motor and cognitive tasks. Football Exercise Program resulted in improved running, coordination, and explosive leg strength performances as well as shorter visual discrimination times in children regularly attending football courses compared with their sedentary peers. On the whole these results support the thesis that the improvement of motor and cognitive abilities is related not only to general physical activity but also to specific ability related to the ball. Football Exercise Programs is assumed to be a “natural and enjoyable tool” to enhance cognitive resources as well as promoting and encouraging the participation in sport activities from early development.

## Introduction

During the last decades, movement development has been intensively researched by a whole approach integrating the growth and the coordination of motor, sensory, and cognitive abilities with respect to the neural maturation in different regions of the brain (Davis et al., 2011). Coherently, a multidisciplinary approach has been employed. This has required the interaction of different disciplines such as kinesiology, developmental psychology and neuroscience, which, historically, have had little contact despite investigating similar issues (Best, 2010).

Currently, there is renewed interest, supported by wide empirical evidence, aimed to show the close relationship between motor development and cognitive functions (Chomitz et al., 2009; Haapala, 2013; Alesi et al., 2014a). Indeed the causal link between regular physical activity and brain growth in the prefrontal cortical area (Best, 2010) represent the crucial point. This close relationship finds a plausible explanation in the *executive function hypothesis* assuming that exercise training sessions influence a significant rise in gray matter volume and a prolonged increase of myelination and connectivity between age 7 and young adulthood in pre-frontal and frontal cortex. The refinement of cortical network in this area improves children’s components of executive abilities: speed and accuracy of processing, strategy employ, working memory, and response inhibition (Diamond, 2011; Diamond and Lee, 2011). Human movement is an expression of human personality, because it associates psychological components and physical structures. A motor skill is a learned sequence of movements that combine to produce a smooth, efficient action in order to become expert in specific goal-directed tasks (Farhat et al., 2015). A child must receive many opportunities to have motor experiences and improve motor coordination in order to develop successfully motor skills. In turns, motor coordination is the harmonious functioning of body parts that involve movements, including gross motor movements, fine motor movements, and motor planning. The first ones require the use of large muscle groups to perform tasks like walking, balancing, crawling. Much of the development of these skills occurs during early childhood and the performance level of gross motor skill remains unchanged after periods of non-use. Conversely, fine motor skills requires the use of smaller muscle groups to perform tasks that are precise in nature. Activities, like kicking the ball, playing the piano or writing, related with technical skills, are examples of fine motor skills. Finally motor planning is the ability of the brain to plan and execute a sequence of unfamiliar actions or non-habitual tasks (Stratton et al., 2004; Von Hofsten, 2004; Libertus and Landa, 2013).

So researchers and practitioners have increasingly worked to clarify the reason that physical and sport activities are suitable to influence cognitive development in children. It’s largely recognized how complex motor tasks, involving coordinative exercises, are more closely related to children’s cognitive functioning than simple motor tasks (Planinsêc and Pisôt, 2006; Alesi et al., 2014b). Coordinative abilities are traditionally recognized to stimulate the activation of the cerebellum effecting on working memory as well as on the speed and accuracy of attention tasks (Budde et al., 2008). In this perspective, football has revealed to be a physical activity able to improve both motor and cognitive growth respectively (Vestberg et al., 2012). Kun and Toth (2012) carried out research on the cognitive resources of young football players. They stated that children playing football need to order, classify and group information they perceive; consequently they improve their ability to understand the connections between information and apply formal thinking. So at the very early age football players are not only able to react to the actions during the game and use technical rules, but they train tactical-cognitive abilities, which in turns, contribute to improve their cognitive profile.

Football stimulates not only simple technical elements during training sessions but also motor and cognitive growth, specifically attention abilities. Players are required to respond quickly and accurately to the actions during the game and continuously evaluate and monitor the match situations. Simultaneously they need to keep in mind all the elements that have already occurred. Therefore, sophisticated levels of thinking are required by the special movements of football; the player analyzes the changing play situations by using his perceptual abilities and realizes them by using his cognitive abilities; consequently, he decides and executes his decision by using his technical and kinetic abilities (Helsen and Starkes, 1999; Jansen et al., 2012). Already at 9 years-old football players show reduced reaction times and more increased decision-making abilities than their sedentary peers (Chang et al., 2013).

In light of these theoretical considerations, the aim of this study was to investigate whether a Football Exercise Program taken over a 6-months period would improve motor and cognitive performances of children aged 9 < 10 years. It was hypothesized that children regularly attending the football course obtained higher gains on motor skills such as running, coordination skills and explosive leg strength as well as cognitive abilities such as visual discrimination times and visual selective attention times, than their sedentary peers.

A further aim was to assess the link between motor skills and cognitive abilities in children practicing football and sedentary children. It was hypothesized a closer link between the above-mentioned abilities in the group composed of children regularly attending the Football Exercise Program.

## Materials and Methods

### Description of Parent Study Recruitment

Forty-six children with age 9.10 years (range: 7–11; *SD* = 1.29) voluntarily participated in this study. The children were subdivided into two groups: first group (Group 1) was composed by 24 (53.2%) children regularly attending football courses and second group (Group 2) was composed of 22 (46.8%) sedentary children; anthropometric measures showed in **Table 1.** Both groups were composed of males, being football a predominant masculine sport. Football children were recruited in their gym and sedentary children in their schools. Prior to the start of the study, each participant’s parents provided written informed consent. Moreover, appropriate local ethics committee approval was obtained from the University of Palermo.

**Table 1:** Descriptive statistics for age and BMI in the football and sedentary groups.

Subjects	Age (years)	Weight (kg)	Height (cm)	BMI (kg⋅m^-2^)
Football group (*n* = 24)	8.78 ± 1.13	35.21 ± 10.98	1.34 ± 8.33	16.04 ± 8.54
Sedentary group (*n* = 22)	9.41 ± 0.96	42.48 ± 10.17	1.40 ± 9.83	21.10 ± 3.76


*Anthropometric measures for both groups related to: age – body weight – body height – body max index (BMI).*

### Procedures and Measures Relevant for the Current Study

A longitudinal method research was employed. Specifically, this research design included: the pretest evaluation (T0) aimed at measuring motor and cognitive abilities; the Football Exercise Program; the post-test evaluation (T1) after 6 months of training in which tests for motor and cognitive abilities were repeated. At the pre-test and at the post-test phases, two measurement sessions were realized: (1) physical/motor session; (2) cognitive session. The second session was carried out over 2 weeks. Children were assessed in the school and gym context twice, the pretest given in November, and the post-test given in May in the same year at the same day-time (2–4 p.m.) to avoid circadian effects (Ammar et al., 2015). At the pretest phase, motor and cognitive abilities were assessed. After the pre-test, the subjects of Group 1 took part in a structured Football Exercise Program, which included several exercises to improve their coordination with an exciting and enjoyable approach, rather than focusing on competition or skill enhancement. Sedentary children didn’t practice any sport activity. After the Football Exercise Program, the post-test phase included a re-evaluation of motor and cognitive abilities.

#### Anthropometric and Motor Assessment

**FIGURE 1:**
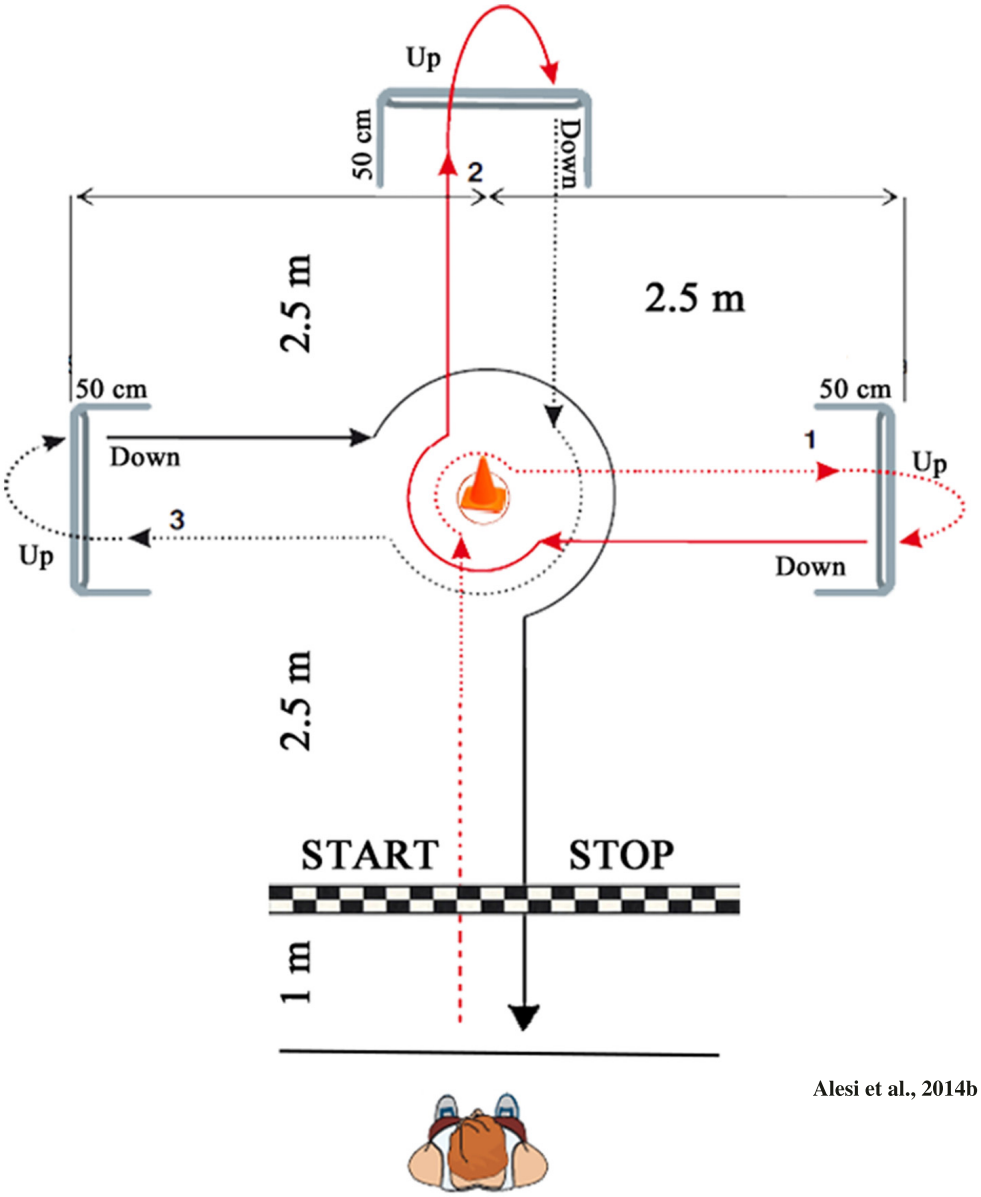
**Agility test**.

Height and body weight (BW) were measured according to standardized practices recommended at the Airlie Conference (Lohman et al., 1988). Body mass index (BMI) was calculated as BW divided by height squared (kg⋅m^-2^). Motor skills were tested by a battery including 20-m Sprint test, Agility test and Standing board jump test. The 20-m Sprint test assessed children’s running skills. Children were asked to linear sprint on a 20 m flat fixed with marker cones (Hammami et al., 2015) and the time was expresses in second. The Agility test (Alesi et al., 2014b) assessed children’s coordinative skills along a circuit with hurdles (**Figure 1**): from the starting line the child had to overcome a central cone, turn right and reach the first obstacle of 50-cm in height, overcome with a leap and immediately go under the same in the opposite direction, return to the central cone and repeat the procedure in the four cardinal directions. Children were asked to complete this circuit as fast possible and without errors. The score was- expressed in second calculated by stop-watch (HS-80TW-1EF, 1/1000 s – Casio Europe).

The Standing board jump test assessed the explosive leg strength. Children were asked to jump horizontally as far as possible landing with both feet from a standing position (Padulo et al., 2014). The score was the longest of three trials. The jump was repeated if children failed or took a step at take-off. For all tests the equipment included meter, clipboard, recording sheet, and pencil ready for the assessment.

#### Cognitive Assessment

Cognitive abilities were tested by the Visual Discrimination test and Visual Selective Attention test derived from the Italian Neuropsychological Battery BVN 5-11 (Bisiacchi et al., 2005). The Visual Discrimination test assessed the ability to identify correctly stimuli by finding likenesses and differences in stimuli. Given a target, children were asked to recognize all similar figures within a matrix of nine shapes. The underlying processes of perceptual organization were details recognition in visual images, perceptual grouping, figure-ground segregation and global-local processing. Frequent errors were reversals, omissions, and additions in the figure recognition. The score was the execution time indicated in seconds.

The Visual Selective Attention test was a paper-and-pencil canceling task, an object decision task aimed at evaluating the object knowledge and recognition. Children were asked to select and cross out targets as quickly as possible (within 1 min). Targets were geometrical shapes represented by squares with two lines inside and were embedded in a frame containing 64 randomly positioned items of which 52 are distractors. Distractors were squares sharing 0 or 1 feature with the target. At first a sample was presented to children to ensure that the instructions were clear. The score was the execution time indicated in seconds.

#### Football Exercise Program

The Football Exercise Program consisted in 75-min sessions, twice a week with 2 days in between, for 6 months. Each session involved the following stages: – for 10 min, the central training period divided into three parts (individual skills, technique or/and 1 vs. 1 man, opposed games 3 vs. 3/5 vs. 5) exercises for 60 min, and cool down for 5 min (**Table 2**). The warm-up phase included a 4-min dribble while walking, circular passing with jogging or kicking the ball against wall, and 6-min of a structured play to improve the motor awareness, control, and coordination without stretching (Chaouachi et al., 2015). The main exercise phase was arranged to allow children to progress from simple to complex and from low to high intensity. These included exercises to improve individual play techniques and develop the creativity of youth players. The cool-down phase consisted in stretching exercises while discussing the main course themes. Sessions were multipurpose in order to develop different aspects of the game in the same session. The exercise program was performed by a soccer-certified teacher.

**Table 2:** Football Exercise Program.

Phase	Activity	Minutes
Warm-up	∘ 4-min dribbles while walking	
	∘ 6-min of a structured play	10
Training	∘ Individual skills	
	∘ Technique or/and 1 vs. 1 (man to man)	50
	∘ Opposed games 3 vs. 3/5 vs. 5	
Cool-down	∘ Stretching exercises while discussing the main course themes	5

*Exercise program divided for: warm-up, training, and cool-down; related to time in minutes.*

#### Data Analysis

All data were expressed as mean values with standard deviation (mean ± SD). These were analyzed by a two-way repeated measures analyses of variance (ANOVA) and adjusted using the Bonferroni’s correction. The within factor was the time with two levels (pre and post-training) and the between factor was the training design with two levels. To assess reliability and variability of the measures, we calculated coefficient of variation [CV = (SD/mean) 100]. Univariate ANOVA were performed to investigate the interaction between/within both groups for motor and cognitive abilities Fisher “F” value was calculated to assess. To interpret effect sizes (ESs) for statistical differences in the ANOVA, we used partial eta square classified as small (0.01 < η^2^ ≤ 0.06), medium (0.06 < η^2^ ≤ 0.14) and large (η^2^ > 0.14; Cohen, 1988). The independent variable was the sport activities/sedentary life style, whilst the dependent ones were motor abilities such as running skills, coordination skills and explosive legs strength and cognitive abilities such as times of visual discrimination and attention. Pearson’s correlation test was performed to assess the association between motor and cognitive skills. For all statistical tests the level of significance was set at *p* < 0.05. The SPSS Software (Version 20 for Windows) was adopted.

## Results

Mean, standard deviation and percentages of score gains of the all variables are presented in **Table 3.** There was no difference between groups at baseline conditions for cognitive and physical test. With concern motor abilities preliminary analyses revealed that football and sedentary groups were equivalent at the pretest evaluation of Agility test [*F*_(1,44)_ = 0.100; *p* = 0.722; $\eta_{p}^{2}$ = 0.002] and Standing board jump test [*F*_(1,44)_ = 1.829; *p* = 0.255; $\eta_{p}^{2}$ = 0.040]. With concern cognitive abilities preliminary analyses revealed that football and sedentary groups were equivalent at the pretest evaluation of times of Visual Discrimination [*F*_(1,44)_ = 1.615; *p* = 0.107; $\eta_{p}^{2}$ = 0.035] and times of Attention [*F*_(1,44)_ = 3.558; *p* = 0.070; $\eta_{p}^{2}$ = 0.073]. At post-test significant differences between the football group and the sedentary group revealed in Sprint test [*F*_(1,44)_ = 10.970; *p* < 0.01; $\eta_{p}^{2}$ = 0.200], Agility test [*F*_(1,44)_ = 27.526; *p* < 0.001; $\eta_{p}^{2}$ = 0.385], Standing board jump test [*F*_(1,44)_ = 15.317; *p* < 0.001; $\eta_{p}^{2}$ = 0.258] and Visual Discrimination test [*F*_(1,44)_ = 4.116; *p* < 0.05; $\eta_{p}^{2}$ = 0.086].

**Table 3:** Performance variables studied in both groups.

Groups	Variable	Pre-test			Post-test			*F*		Percentage score gains
					
		*M*	*SD*	CV	*M*	*SD*	CV	*F*-value	*p*	
Football group	Agility (sec)	21.46	6.52	30.38	15.49	2.39	15.43	17.685	<0.05	27.82%
(*n* = 24)	Sprint (sec)	4.29	0.40	9.32	4.07	0.24	5.89	5.332	<0.05	5.13%
	SBJ (cm)	118.05	20.18	17.09	125.54	17.81	14.09	1.858	0.179	6.34%
	TVD (sec)	58.64	16.08	27.42	47.08	9.20	19.54	9.420	<0.05	19.71%
	TVSA (sec)	57.96	11.49	19.82	54.74	15.37	28.08	0.692	0.410	5.56%
Sedentary group	Agility (sec)	20.86	6.15	29.48	19.56	2.8	14.31	0.820	0.370	6.23%
(*n* = 22)	Sprint (sec)	4.63	0.31	6.69	4.37	0.35	8.01	6.754	<0.05	5.62%
	SBJ (cm)	110.13	19.43	17.64	104.83	18.05	17.21	0.880	0.354	4.81%
	TVD (sec)	65.91	22.87	34.70	56.77	21.35	37.61	1.876	0.178	13.87%
	TVSA (sec)	76.63	48.28	63.01	61.05	20.68	33.87	1.961	0.169	20.33%


*Values as mean ± SD, coefficient variation (CV), Fisher (F) values and percentage score (%) gains for: Agility test, Sprint test, Standing Board Jumping test (SBJ), Times of Visual Discrimination (TVD), Times of Visual Selective Attention (TVSA).*

Specifically, in the football group significant improvements were found from pretest to post-test in scores at the 20 m Sprint [*F*_(1,44)_ = 5.332; *p* < 0.05; $\eta_{p}^{2}$ = 0.104] and the Agility test [*F*_(1,44)_ = 17.685; *p* < 0.01; $\eta_{p}^{2}$ = 0.278]. On the contrary, in the sedentary group weren’t found differences in scores at the Agility test [*F*_(1,44)_ = 0.820; *p* > 0.05; $\eta_{p}^{2}$ = 0.019] and Standing board jump test [*F*_(1,44)_ = 0.880; *p* = 0.354; $\eta_{p}^{2}$ = 0.021]. Only at the Sprint test significant differences were found [*F*_(1,44)_ = 6.754; *p* < 0.05; $\eta_{p}^{2}$ = 0.013]. Moreover in the football group significant improvements were found from pretest to post-test in scores at the times of Visual Discrimination [*F*_(1,44)_ = 9.420; *p* < 0.05; $\eta_{p}^{2}$ = 0.167].

Looking at percentage score gains gives suggestion of the size of the improvements in motor skills and speed of visual discrimination. In the trained group, times of execution of coordinative circuit improved showing a decrease of 27.82% from pre-test to post-test, whilst in the control group it decreased of 6.23%. Moreover, in the football group, performances in Standing board jump task improved of 6.34% and in the 20 m Sprint of 5.13%. Increases in the control group were smaller; performances in the Standing board jump task improved of 5.62% and in the 20 m Sprint of 5.62%. The time of visual discrimination decreased of 19.71% in the football group and 13.87% in the sedentary group. Finally, correlation analyses showed in the football group positive significant correlations between running skills and Visual Discrimination times or Attention times (see **Table 4**). Moreover, Standing board jump was significantly and negatively related to Visual Discrimination times.

**Table 4:** Relationship between performance variables in Football Group.

		(1)	(2)	(3)	(4)	(5)
Agility	(1)	1				
Sprint	(2)	0.743ˆ**	1			
SBJ	(3)	-0.677ˆ**	-0.757ˆ**	1		
TVD	(4)	0.177	0.416ˆ*	-0.458ˆ*	1	
TVSA	(5)	0.317	0.473ˆ*	-0.225	0.236	1


*Pearson correlations between Agility, Sprint, Standing Board Jumping (SBJ), Times of Visual Discrimination (TVD), and Times of Visual Selective Attention (TVSA) in Football Group. Significant value was showed with ^∗^*p* < 0.05 and ^∗∗^*p* < 0.01.*

In the sedentary group the only significant correlation was found between coordinative skills and Visual Discrimination times (see **Table 5**).

**Table 5:** Relationship between performance variables in Sedentary Group.

		(1)	(2)	(3)	(4)	(5)
Agility	(1)	1				
Sprint	(2)	0.467ˆ*	1			
SBJ	(3)	-0.738ˆ**	-0.762ˆ**	1		
TVD	(4)	0.478ˆ*	0.134	-0.414	1	
TVSA	(5)	0.284	-0.057	-0.303	0.664ˆ*	1


*Pearson correlation between Agility, Sprint, Standing Board Jumping (SBJ), Times of Visual Discrimination (TVA), and Times of Visual Selective Attention (TVSA) in Sedentary Group. Significant value was showed with ^∗^*p* < 0.05 and ^∗∗^*p* < 0.01.*

## Discussion

Our Football Exercise Program resulted in improved running skills and coordination skills and explosive legs strength as well as shorter times of visual discrimination in children regularly attending football courses compared with sedentary peers. First, worthy of note is that at post-test football participants better performed than their sedentary peers with concern their scores in motor tests. At Sprint test, Agility test, and Standing board jump test they showed significant higher skills than sedentary peers, how revealed by large ES for statistical differences between the two groups. Moreover, children regularly attending the Football Exercise Program obtained the largest gain between pre-test and post-test in the agility test, measuring coordinative performance, how shown by the higher ES for statistical differences from baseline to post-test and the decrease of CV from baseline to post-test. This is not unexpected because football improves most speed, rapidity and dynamic balance. Playing football requires that the environment constantly changes and movements have to be continually adapted. This play paradigm involves many motor processing stages like to structure the appropriate motor program: change run speed and direction, jump, kick the ball and tackle (physical contact between players). With growth the body changes adapting to the exercise program and enhancing motor capabilities in those children who constantly practice football. On the other hand, sedentary children have not these stimuli to improve motor capabilities and maintain their starting point (Stratton et al., 2004; Von Hofsten, 2004). So, as hypothesized, the study revealed the value of regular football in increasing speed and coordination skills in children. Football is not only jogging and kicking the ball. It doesn’t need only the interaction of agonist and antagonist muscles but it involves both motor coordination and perceptual skills (Chang et al., 2013).

Moreover we think it is quite interesting to show that with concern the cognitive measures only children regularly attending the Football Exercise Program obtained significant gains at the post-test and the decrease of CV in the Visual Discrimination test. Specifically, our sport children improved their abilities in the times of visual discrimination with medium ES for the gain from baseline to post-test. They showed lower reaction times in the task of details recognition in visual images, perceptual grouping, figure-ground segregation and global-local processing than their sedentary peers. These results support a key role for football activities to improve perceptual organization. Coordinative exercises, such as those stimulated by football, involve significant top–down cognitive control and the ability to ignore automatic behaviors (Diamond and Lee, 2011). These complex coordinative activities revealed to be particularly suitable to stimulate the components of visual-spatial attention such as the visual search which develops markedly during childhood. To be able to select targets from the same stimulus category entails a more serial search. This ability of visual search requires not only the visual selective or focal attention, the peripheral visual acuity in order to move the attentive focus or the ability to divide visual attention when faced to multiple objects, but more sophisticated meta-cognitive strategies. In this way the football arouses the coordination of complex body movements, the employ of strategic behaviors and cooperation with other children and adjustment to continually changing play demands. Further, how Diamond (2015) argues, these benefits to cognitive development stem from activities requiring and challenging executive functions, but also providing enjoyment, pride, a sense of belonging and social support. All these factors together contribute to increase physical skills and self-confidence.

A further aim of our study was to assess the association between motor skills and cognitive abilities. Correlation analyses confirmed the feasibility of our study design. As expected, the football group showed significant associations between running skills, visual discrimination times, and attention times. Conversely, visual discrimination times were negatively related to the performance on standing board jump which is a field test muscle mass and muscle characterization dependent, even on that case as expected. This is consistent with the hypothesis that to train running skills is a way to train indirectly cognitive abilities because football skills involve sophisticated cognitive abilities to anticipate team-mates and opponents’ behaviors as well as use strategies to adapt to changing task demands (Alesi et al., 2014b).

In summary we argue that in our study the improvement of cognitive and motor skills is related not only to general physical activity but also to a specific sport, as well as football, based on the assumption that different sports activities require different sets of skills. A successful player has to show those cognitive abilities called “game intelligence” which involve spatial attention, shared attention, working memory, and metalizing ability (Jansen et al., 2012). The play paradigm of football involves many perceptual, cognitive, and motor processing stages: (1) to perceive the environment by discriminating among several visual-spatial stimuli; (2) to recognize and evaluate the appropriate features; (3) to decide what actions are necessary by mapping them to the assigned response; (4) to structure the appropriate motor program; (5) to execute the chosen action (Battaglia et al., 2013; Wang et al., 2013).

To the best of our knowledge, literature is lacking in studies, which investigated cognitive functioning in children practicing football. The majority of researchers focused on expert or elite adults players in order to train mental abilities able to maximize their sport efficiency (Voss et al., 2010; Vestberg et al., 2012). So the present study contributes additional evidence that suggests the critical role of football practice to favor cognitive development in childhood. The main strength of this study lies in the planning of a Football Exercise Program as a “natural and enjoyable tool” to stimulate and increase cognitive resources. However, this study is not without its limitations. First and foremost, we tested only 46 children. We planned to test larger and different groups to analyze the contribute that different sports can do to children development, e.g., differences between individual and team sports or open and closed skills sport. Second, long-term maintenance of obtained gains need to be evaluated by follow-up at 6 or 12 months.

On the whole, our results have highlighted that practitioners and service providers have to recognize cognitive potentialities stimulated by sport activities and focus into the possibility to plan evidence-based programs aimed at promoting and encouraging the participation in sport activities from early development.

## Conflict of Interest Statement

The authors declare that the research was conducted in the absence of any commercial or financial relationships that could be construed as a potential conflict of interest.
